# Supplementary material for: Elderly Gliobastoma Patients: The Impact of Surgery and Adjuvant Treatments on Survival: A Single Institution Experience
Source: Brain Sci. 2022 May 11;12(5):632. doi: 10.3390/brainsci12050632 (PMC9139732; doi:10.3390/brainsci12050632)
Supplement: Supplementary file 1 [file brainsci-12-00632-s001.zip › brainsci-1687565-supplementary.pdf]

Supplementary Table S1. Baseline characteristics, treatment modalities and outcome of patients according to sex.

	Male	Female	p value
Median age, years	70.0	72.0	0.420
CCI > 3	34/87 (39.1%)	22/48 (45.8%)	0.446
KPS ≥ 70	83/87 (95.4%)	44/48 (91.7%)	0.379
*MGMT*p methylation	47/83 (56.6%)	23/45 (51.1%)	0.550
Exent of resection			
Gross-total resection	16/87 (18.4%)	7/48 (14.6%)	
Subtotal resection	64/87 (73.6%)	38/48 (79.2%)	0.769
Biopsy	7/87 (8.0%)	3/48 (6.3%)	
Complications after surgery	25/87 (28.7%)	11/48 (22.9%)	0.464
Adjuvant Treatment			
6-week RT/TMZ + TMZ	24/87 (27.6%)	9/48 (18.8%)	
3-week RT/TMZ + TMZ	24/87 (27.6%)	13/48 (27.1%)	
RT + TMZ	6/87 (6.9%)	7/48 (14.6%)	
RT alone	10/87 (11.5%)	4/87 (8.3%)	0.468
TMZ upfront	10/87 (11.5%)	4/87 (8.3%)	
Palliation	13/87 (14.9%)	11/48 (22.9%)	
mPFS (months, 95% CI)	8.7 (5.4-12.0)	6.6 (3.5-9.6)	0.498
mOS (months, 95% CI)	10.7 (7.5-13.8)	9.2 (6.0-12.4)	0.523

Abbreviations: CCI, Charlson Comorbidity Index; KPS, Karnofsky Performance Status; *MGMT*p, O(6)-methylguanyl DNA methyltransferase promoter; mOS, median overall survival; mPFS, median progression-free survival; RT, radiotherapy; TMZ, temozolomide.

Supplementary Table S2. Median progression-free survival and overall survival according to Charlson Comorbidity Index within different classes of age.

Age Class	Age-adjusted CCI*	Median Progression-free Survival				Median Overall Survival			
		Months	95% Confidence Interval		p value	Months	95% Confidence Interval		p value
			Lower Bound	Upper Bound			Lower Bound	Upper Bound	
65-69 yrs	≤ 3	8.1	4.7	11.5	0.002	12.9	9.9	15.8	0.006
	> 3	1.0	1.0	1.1		4.4	2.4	6.4	
70-74 yrs	≤ 3	8.2	2.1	14.3	0.950	10.0	8.4	11.6	0.975
	> 3	12.7	/	/		16.2	/	/	
75-79 yrs	≤ 3	4.3	0.1	8.9	/	8.8	7.1	10.5	/
	> 3 †	Not applicable				Not applicable			
≥ 80 yrs	≤ 3	19.8	11.3	28.4	0.494	28.8	/	/	0.736
	> 3	3.6	/	/		4.2	/	/	

Abbreviations: CCI, Charlson Comorbidity Index; yrs, years. The 95% Confidence Interval was not obtained due to small patient sample in cells marked with '/'.

*CCI was modified by subtracting the value attributed to age from the total score, to make comorbidities the only factors included in the index.

† No patient had CCI > 3 in the 75-79-year-old class of age.
